# *Exophiala* Bloodstream Infections in Humans—A Narrative Review

**DOI:** 10.3390/pathogens14070706

**Published:** 2025-07-17

**Authors:** Afroditi Ziogou, Alexios Giannakodimos, Ilias Giannakodimos, Stella Baliou, Andreas G. Tsantes, Petros Ioannou

**Affiliations:** 1 Department of Medical Oncology, Metaxa Cancer Hospital of Piraeus, 185 37 Piraeus, Greece; 2 Department of Cardiology, Tzaneio General Hospital of Piraeus, 185 37 Piraeus, Greece; 3 Departement of Urology, Attikon General Hospital of Athens, 124 62 Athens, Greece; 4 School of Medicine, University of Crete, 710 03 Heraklion, Greece; 5 Laboratory of Hematology and Blood Bank Unit, “Attikon” University Hospital, School of Medicine, National and Kapodistrian University of Athens, 124 62 Athens, Greece; andreas.tsantes@yahoo.com

**Keywords:** *Exophiala*, *Wangiella*, fungemia, bloodstream

## Abstract

**Background:** *Exophiala* spp. are dematiaceous fungi with opportunistic pathogenic potential and a widespread environmental presence. Clinical cases of *Exophiala* spp. fungemia are uncommon. Although rarely encountered in the general population, these organisms are increasingly reported in immunocompromised individuals or those with complex underlying health conditions. **Objectives:** This review seeks to examine all documented human cases of *Exophiala* spp. fungemia, with particular focus on aspects such as epidemiology, microbiological features, resistance patterns, therapeutic approaches and associated mortality rates. **Methods:** A narrative review was conducted using data sourced from the PubMed/MedLine and Scopus databases. **Results:** A total of 19 articles described infections in 32 patients involving *Exophiala* spp. fungemia. The mean patient age was 49.2 years, and 65.6% were male. Central venous catheters emerged as the leading predisposing factor (96.9%). Fever represented the most frequent clinical presentation (50%), followed by organ dysfunction (21.9%). The yeast generally demonstrated susceptibility to voriconazole and itraconazole. Voriconazole was also the most frequently administered antifungal (62.5%), followed by amphotericin (31.3%) and micafungin (28.1%). Overall mortality reached 34.4%, with 25% of deaths specifically caused by the infection. **Conclusions:** Given the potential of *Exophiala* spp. to cause severe fungemia, healthcare professionals, particularly clinicians and microbiologists, should consider this pathogen in the differential diagnosis when black yeast is detected in blood cultures, especially in patients with immunodeficiency or significant comorbidities, to ensure timely and accurate identification.

## 1. Introduction

*Exophiala* is a genus of dematiaceous fungi belonging to the order Chaetothyriales, commonly referred to as black yeasts due to their dark pigmentation resulting from melanin production in the cell wall. These fungi are characterized by their slow growth and morphological plasticity, exhibiting both yeast-like and filamentous forms. Black yeasts and their filamentous relatives are known to cause numerous severe and potentially life-threatening infections in both immunocompetent and immunocompromised individuals. They can also act as opportunistic pathogens in vertebrate hosts [1]. *Exophiala* spp. are typically found in environments like soil, water, decaying organic material and humidifiers [2]. Infections caused by these pathogens often present as skin or subcutaneous lesions that may lead to systemic infections, particularly in immunocompromised individuals [3]. Although systemic infections are extremely rare, they are linked to high morbidity and mortality rates. *Exophiala dermatitidis* is the most clinically significant species within this genus [4]. The thermotolerance and environmental resilience of *E. dermatitidis* contribute to its pathogenicity and persistence, while its slow growth and phenotypic similarity to other fungi often complicate timely diagnosis and treatment [5]. No standardized treatment guidelines currently exist for *Exophiala* fungemia; consequently, most cases are managed empirically while pending antifungal susceptibility results [6]. Mortality rates associated with this infection are relatively elevated, with patient outcomes primarily dependent on the individual’s underlying health status.

This study primarily aims to review all reported cases of fungemia caused by *Exophiala* species in humans, with a special focus on epidemiological patterns and associated mortality. It also seeks to characterize the microbiology, antifungal susceptibility and therapeutic approaches to this infection while addressing gaps in knowledge regarding risk factors and treatment strategies, thereby contributing to the limited literature on this emerging pathogen.

## 2. Materials and Methods

### 2.1. Search Strategy and Inclusion and Exclusion Criteria

This narrative review aims to compile and summarize all available cases concerning human bloodstream infections caused by *Exophiala* species. The main goal was to provide an overview of the epidemiology and associated mortality rates. Secondary objectives included information on predisposing risk factors, clinical presentation, microbiological characteristics and therapeutic approaches. A comprehensive search was performed in the PubMed/Medline and Scopus databases by two independent reviewers (A.Z. and A.G.) up to 10 June 2025, using a structured data extraction form. The search strategy included the following terms: (“Exophiala” OR “Wangiella”) combined with (“fungemia” OR “bloodstream”). Any disagreements during the selection process were resolved by consulting a senior reviewer (P.I.). Eligible studies were those presenting original human data, including case reports, case series and cohort studies that provided epidemiological or clinical outcome information on *Exophiala* spp. infections in patients of all ages, as well as paediatric patients. Only English-language publications were included. Reviews, systematic reviews, animal studies and articles lacking full-text access or those missing key data on mortality or epidemiology were excluded. To ensure a comprehensive review, the reference lists of all selected articles were also screened for additional relevant publications.

### 2.2. Data Extraction and Definition

For each study included in this review, data on the year of publication, study design, country of origin and patient characteristics, including age and gender, were extracted. Additional information collected encompassed relevant medical background, specifics of the infection, such as complications or symptom duration and microbiological details, including the identified pathogen, antifungal resistance patterns, treatment approaches and patient survival outcomes. The connection between the initial infection and mortality was reported based on the interpretations provided by the original study authors.

## 3. Results

### 3.1. Included Studies’ Characteristics

**Figure 1:**
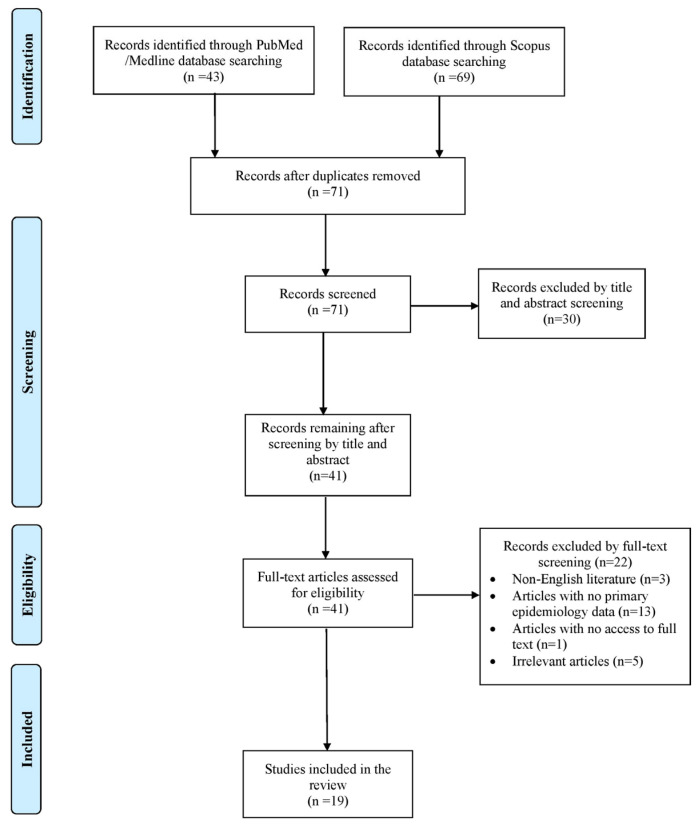
Trial flow of this review.

Initially, 112 articles were identified through searches of the PubMed/Medline and Scopus databases. Following the elimination of duplicates, a thorough screening of records and the application of the snowball method, only 19 articles met the established inclusion criteria and were ultimately chosen for detailed review [4,6,7,8,9,10,11,12,13,14,15,16,17,18,19,20,21,22,23]. These publications collectively described 32 individual patient cases. The article selection process is outlined in the flowchart shown in Figure 1. Among the documented cases, 18 (56.3%) were reported from North America, 8 (25%) from Asia, 5 (15.6%) from Europe and 1 (3.1%) from South America. All selected studies were case reports and case series. Table S1 shows the characteristics of the included studies.

### 3.2. Epidemiology of Exophiala spp. Fungemia

The mean age of individuals diagnosed with *Exophiala* spp. fungemia was 49.2 years, with ages ranging from newborns to 81 years. Among the 32 patients, 21 (65.6%) were male. Regarding medical background and predisposing factors, 31 patients (96.88%) had central venous catheters, while 25 patients (78.1%) were diagnosed with malignancy. Of note, 11 individuals (37.4%) were receiving chemotherapy, and in 8 cases (25%), the malignancy was hematologic. Moreover, 10 patients (31.3%) were immunocompromised, while 8 (25%) exhibited neutropenia. Four individuals (12.5%) had recently undergone hematopoietic cell transplantation (HCT) or were receiving total parenteral nutrition (TPN). Additionally, three patients (9.4%) had been subjected to intubation or administered antibiotics within the past three months. Finally, one individual (3.1%) was diagnosed with HIV, and another infant was born prematurely. A summary of the demographic and clinical features of *Exophiala* spp. fungemia cases is presented in Table 1.

### 3.3. Antifungal Resistance and Microbiology of Exophiala spp. Fungemia

*Exophiala* species were detected in both blood and CVC cultures in 21 patients (65.6%), while in 8 (25%) and 3 patients (9.4%), the pathogens were isolated only from blood cultures and only from CVC cultures, respectively. In one patient (3.2%), the pathogen was identified in pleural fluid specimens. The most frequently isolated species was *Exophiala dermatitidis*, found in 30 cases (93.8%), followed by *E. oligosperma* and *E. jeanselmei*, each identified in one patient (3.2%). No other species were identified. Polymicrobial infections were observed in five patients (15.6%); two of these cases involved co-infection with *Rhodotorula mucilaginosa*. Pathogen identification primarily relied on molecular diagnostics, with matrix-assisted laser desorption/ionization time-of-flight (MALDI-TOF) mass spectrometry successfully confirming the organism in seven cases (58.3%, based on available data). Additionally, DNA sequencing as well as GenBank BLAST analysis contributed to the identification of two cases each (16.7%, based on available data). Antifungal susceptibility testing was performed in only 12 patients (37.5%). Details on antifungal resistance profiles are provided in Table 2. In each patient, a different combination of antifungals was tested. Interestingly, in all cases tested for resistance to voriconazole, itraconazole or posaconazole, all pathogens were found to be sensitive.

### 3.4. Clinical Presentation of Exophiala spp. Fungemia

*Exophiala* spp. most commonly presented with fever in 16 cases (50%) and organ dysfunction in 7 cases (21.9%). The system most frequently affected was the respiratory, resulting in respiratory failure in five of these cases (15.6%). Moreover, two cases of multiple organ failure (6.3%) and one case of renal failure (3.1%) were described. Six individuals (18.8%) required hospitalization in the intensive care unit (ICU) while four individuals (12.5%) developed sepsis or hemorrhagic diathesis, and one patient (3.1%) developed septic shock. The symptom duration varied between 0 days (acute onset of fungemia) and 6 months.

### 3.5. Treatment and Outcome of Exophiala spp. Fungemia

According to the collected data, all patients received antifungal therapy. Voriconazole was the most commonly administered antifungal, given to 20 patients (62.5%), followed by amphotericin B and micafungin, used in 10 (31.3%) and 9 patients (28.2%), respectively. Itraconazole was used in six cases (18.8%), while fluconazole and anidulafungin were each given to four (12.5%) and one patient (3.2%). In 16 patients (50%), antibiotics were also administered before the establishment of the final diagnosis. Surgical procedures combined with antifungal therapy were performed in only one patient (3.2%) and consisted of abdominal and adjacent necrotic intestinal infected tissue resection en bloc.

For those who survived, the median length of treatment was 42 days. The overall mortality rate observed was 34.4% (11 of 32 patients), with deaths specifically linked to *Exophiala* spp. fungemia accounted for 25% (8 of 32 patients). The mortality rate among patients with cancer was estimated at 32% (8 out of 25), 40% (4 out of 10 cases) among immunosuppressed individuals and 37.5% (3 out of 8 cases) among patients with neutropenia. Among patients subjected to HCT, the mortality rate was 75% (three out of four cases), while among patients receiving TPN, it was calculated at 50% (two out of four cases).

## 4. Discussion

This narrative review summarizes the features of bloodstream infections caused by *Exophiala* species, with data derived from multiple studies that provide comprehensive information on epidemiology, microbiology, clinical manifestations, treatment approaches and patient outcomes. To our knowledge, this is the first review that tries to collect knowledge on the identification and management of this rare entity. Among *Exophiala* species, *Exophiala dermatitidis* was the most frequently identified. Voriconazole was the predominant antifungal agent used for treatment. Additionally, this review emphasizes that the overall mortality rate associated with *Exophiala* infections was elevated.

Due to the limited number of reported cases of *Exophiala* spp. infections in the current literature, an accurate determination of their epidemiological profile remains a challenge [13]. Diagnosis of this rare entity mainly relies on the physician’s clinical suspicion of the disease, and thus, the physician should be aware of its existence. In this review, most cases occurred in male patients, with a median age of 58 years. Notably, the majority of cases were reported from Europe and North America, followed by Asian countries. Only one case was documented in South America. The higher number of *Exophiala* fungemia cases reported in European and North American countries may not necessarily reflect a true higher incidence but rather a combination of more advanced diagnostic capabilities, more comprehensive surveillance and reporting systems, as well as higher use of CVCs and immunosuppressive therapies in these countries [18,20,24]. Moreover, healthcare-associated outbreaks of *Exophiala* fungemia due to contaminated medical products or devices have contributed to the higher number of patients in these regions. The low number of cases in Asia and South America, as well as the absence of reported cases in Africa, may be attributed to underdiagnosis of *Exophiala* infections due to limited laboratory capabilities, lack of awareness or lower rates of publication in international journals. Nevertheless, the limited number of studies conducted globally hinders the ability to draw definitive epidemiological conclusions regarding *Exophiala* bloodstream infections.

The genus *Exophiala* comprises over 30 species; these pathogens are melanized fungi commonly referred to as “black yeasts” due to the production of melanin in their cell walls [25]. They are characterized by both yeast-like cells and septate hyphae, a feature typical of dematiaceous fungi [1]. A notable trait of *Exophiala* spp. is their polymorphic growth; colonies initially appear as slow-growing, moist, yeast-like structures and later transition into filamentous forms [26]. Numerous clinically relevant *Exophiala* species, such as *E. dermatitidis*, are thermotolerant, capable of growing at temperatures between 37 °C and 42 °C. This thermotolerance supports their ability to cause systemic infections, particularly in immunocompromised hosts [27]. *Exophiala* spp. are ubiquitous saprobes isolated from several environmental sources, including soil, decaying organic matter, water and high-humidity settings such as steam baths, dishwashers and humidifiers [2,26]. Their infectious potential as opportunistic pathogens is attributed to this environmental adaptability. Moreover, these species are notable for their resilience; melanin production enhances resistance to environmental stressors such as extreme temperatures, pH shifts, oxidative stress, heavy metals and even hydrocarbons, thus further enhancing their infectious potential [28].

*Exophiala* spp. exhibit remarkable adaptive plasticity, enabling the pathogens to cause a diverse spectrum of infections that vary based on the route of entry and the host’s immune status [16]. *Exophiala* spp. have frequently been reported as a respiratory tract opportunistic pathogen in individuals with cystic fibrosis and have occasionally been implicated in cases of fungal pneumonia and pulmonary phaeohyphomycosis [24]. Asymptomatic colonization of the gastrointestinal tract has also been reported. Localized infections typically result from traumatic inoculation of the skin, without subsequent dissemination to deeper tissues. Alternatively, infection may occur through inhalation and, more rarely, through haematogenous spread [29]. Once introduced into the bloodstream, *Exophiala* spp. can lead to systemic and disseminated infections, frequently displaying neurotropic behaviour with involvement of deep organs, such as the brain, lungs and heart. Although invasive infections caused by *Exophiala* spp. are uncommon, they may also occur during outbreaks associated with direct iatrogenic inoculation [1,30].

Concerning predisposing risk factors, the presence of CVCs was identified as the most frequently reported condition among affected individuals, as evidenced by the findings of this review. Interestingly, with the exception of only one case, CVCs were present in all other patients included in the present study. The development of *Exophiala* fungemia is facilitated by CVCs since they bypass the body’s primary defence mechanisms, such as the skin or mucosal barriers, providing direct access into the bloodstream and facilitating fungal entry, especially in immunocompromised patients [13,20]. *Exophiala* spp. also have the capacity to form biofilms on catheter surfaces; these biofilms protect the fungi from host immune responses, increase resistance to antifungal agents and serve as a persistent reservoir for bloodstream infection [31]. Moreover, given the pathogens’ opportunistic nature, in hospital settings, they can contaminate water sources, medical equipment or surfaces. As a result, CVCs, especially if improperly handled or maintained, may become colonized during insertion or use [32]. Extended use of CVCs increases the duration of exposure to potential contaminants and, thus, the possibility of biofilm formation, especially in patients in ICU or with cancer. In a study by Hagiya et al., involving 29 patients with *Exophiala* fungemia, 79.3% had a central venous catheter in place at the time of diagnosis—highlighting how extended catheterization enhances infection risk [17]. A history of malignancy and immunosuppression also emerged as notable risk factors among patients included in this review. Various types of cancers, particularly hematologic, disrupt normal immune cell function or cause cytopenias, leading to impaired neutrophil activity or reduced T-cell–mediated immunity. This immunosuppressed state creates a vulnerable host environment for opportunistic fungi like *Exophiala* spp. [32]. In this study, among infected patients with cancer, 32% (8 out of 25) of cases had hematologic malignancies. Additionally, chemotherapy administration commonly results in neutropenia and mucosal barrier damage, facilitating translocation of fungi into the bloodstream and contributing directly to increased risk of fungemia [33]. Generally, radiation, chemotherapy or tumor burden can damage skin and mucosal surfaces, easing fungal entry; in this manner, *Exophiala* spp. can exploit damaged epithelial surfaces and provoke systemic infection [19].

Another potential predisposing factor for *Exophiala* fungemia is HCT. The present review includes two cases of autologous and one case of allogenic HCT, as well as one case of umbilical cord blood transplantation [6,7,17,19]. HCT induces severe and prolonged immunosuppression since recipients, especially allogenic, undergo intensive immunosuppressive regimens, and the development of neutropenia is common post-HCT [34]. Immunosuppressive agents used to prevent or treat graft-versus-host disease further impair immune responses, especially cell-mediated immunity, which is crucial for defence against fungi. Moreover, frequent broad-spectrum antibiotic administration in these patients disrupts the normal microbiome and creates an ecological niche where fungal organisms can proliferate and invade the bloodstream [35]. TPN may also predispose patients to *Exophiala* fungemia through several mechanisms involving catheter use, nutrient-rich infusions and immune suppression. TPN requires long-term use of CVCs, enabling *Exophiala* bloodstream fungal infections, given the species’ ability to form biofilms on synthetic materials [36]. TPN solutions are rich in glucose, amino acids and lipids, which promote fungal growth. In cases of aseptic technique during preparation or administration, these solutions can serve as ideal media for fungal proliferation [37]. TPN reduces enteral feeding, leading to atrophy of the gut mucosa and loss of mucosal immune regulation [38]. Finally, intubation may constitute another risk factor for the development of *Exophiala* fungemia; three patients included in the present review had been subjected to intubation prior to the infection. Endotracheal tubes and ventilators potentially enable biofilm formation, in which *Exophiala* spp. proliferate. Colonization of the respiratory tract may become a source for dissemination, especially if invasive devices like CVCs are also present. However, intubation alone is not a direct risk, but part of a cluster of factors, including critical illness, CVC or ventilator biofilms, that collectively create the ideal environment for *Exophiala* to invade [39,40]. A case report by Mpakosi et al. demonstrated that fungemia develops in patients with intubation and multiple other high-risk conditions [4].

Identification of *Exophiala* species is challenging, as the majority of microbiology laboratories lack access to advanced molecular tools like genetic sequencing. The diagnostic process for *Exophiala* spp. infections typically begins with microscopic examination, wherein the organism may exhibit either yeast-like or hyphal morphological forms [41]. The identification of sclerotic bodies is particularly indicative of *E. dermatitidis* involvement in cases of chromoblastomycosis [42]. Colonies of *Exophiala* species microscopically appear moist and yeast-like in the early stages, progressively developing dark green to olive-black pigmentation as they mature [43]. Additionally, phenotypic features that distinguish these *fungi* from other species include their capacity to grow at 42 °C, their inability to assimilate nitrite or nitrate, and the absence of annelids upon microscopic examination [14]. *Exophiala* spp. identification relies on morphology and, thus, can often become challenging due to their pleomorphic nature. Molecular techniques, such as MALDI-TOF mass spectrometry or sequencing of the internal transcribed spacer (ITS) regions of ribosomal DNA, have become essential for accurate species-level identification in clinical mycology [10,25,27]. In the present review, MALDI-TOF mass spectrometry emerged as the most commonly applied method for the identification of *Exophiala* species. In accordance with our findings, recent studies have also demonstrated that MALDI-TOF mass spectrometry can reliably identify these microorganisms [16,31]. In a study by Kumar et al., in order to establish definitive pathogen identification, sequencing was performed on a representative isolate obtained from blood, targeting both the ITS region and the D1/D2 domain of the large ribosomal subunit [14]. Sequencing of the ITS1 region is generally recommended for differentiating closely related black yeast species belonging to the same genus. The application of genetic sequencing for the detection of *Exophiala* spp. remains insufficiently explored in the current literature, highlighting the need for further research to generate comprehensive data. Given the high cost and limited accessibility of such advanced techniques, accurate identification should incorporate biochemical and cultural characteristics specific to the pathogen. Blood cultures were performed for all included patients, yielding both biochemical and cultural data. As a result, a positive fungal culture may serve as the initial laboratory indicator, prompting the application of more advanced and innovative molecular diagnostic techniques.

There are currently no standardized or widely recognized antifungal susceptibility breakpoints for *Exophiala* species, as comprehensive studies assessing their susceptibility patterns have not been conducted [44]. As a result, the antifungal susceptibility data discussed in this review are largely based on information reported in individual case studies. *Exophiala dermatitidis* has generally demonstrated high minimum inhibitory concentrations (MICs) for fluconazole and 5-fluorocytosine, indicating reduced susceptibility. In contrast, amphotericin B, itraconazole, voriconazole and posaconazole have shown greater in vitro efficacy against this pathogen [45]. In the present review, isolates generally exhibited susceptibility to voriconazole, itraconazole, posaconazole and amphotericin while demonstrating comparatively higher resistance rates for fluconazole and echinocandins. Of note, the highest resistance rate was observed for echinocandins, especially micafungin (100%). Given the lack of clear understanding of the specific resistance mechanisms exhibited by these pathogens, further research is essential to elucidate the underlying factors contributing to their antifungal resistance. Moreover, the development of standardized criteria for interpreting susceptibility testing results is critical to ensure accurate and reliable assessments of antifungal sensitivity.

The clinical management of *Exophiala* spp. infections presents considerable difficulties, largely due to the absence of formally established antifungal treatment guidelines. In cases of systemic infection, the initiation of prompt and intensive antifungal therapy is strongly advocated to prevent progression to severe or potentially fatal disease [44]. It is advisable to conduct antifungal susceptibility testing to guide therapeutic management, prioritizing agents with the lowest MICs and optimal pharmacokinetic properties for penetration at the site of infection. Although echinocandins exhibit antifungal activity, monotherapy with this class of agents appears suboptimal and may not provide sufficient clinical efficacy in the context of fungemia [16]. Voriconazole is a second-generation triazole that functions by inhibiting cytochrome P450 14α-demethylase, an enzyme critical for the conversion of lanosterol to ergosterol. This inhibition disrupts ergosterol synthesis, an essential component of the fungal cell membrane, thereby impairing fungal cell integrity and growth [46]. In clinical practice, voriconazole has demonstrated favourable outcomes when administered at therapeutic levels guided by drug monitoring. However, the definitive susceptibility breakpoint for *Exophiala* spp. remains under investigation, and it is also crucial to accurately specify the species prior to initiating voriconazole therapy [46,47]. In cases of systemic infection, the addition of amphotericin B to the treatment regimen may also be beneficial. Posaconazole, presenting the broadest spectrum antifungal activity among oral antifungal agents, may also be considered for managing systemic infections [14]. The duration of therapy varies according to the severity of clinical manifestations. In this review, treatment courses ranged from 4 days to 6 months, particularly in cases presenting with severe comorbidities, such as HIV [20]. Surgical procedure was conducted in only one case, alongside antifungal therapy, and consisted of abdominal and adjacent necrotic infected intestinal tissue resection en bloc [21].

*Exophiala dermatitidis* infections often result in severe systemic disease, with a mortality rate approaching 80% as described in the literature [10]. In the present study, mortality rates associated with *Exophiala* spp. fungemia were also relatively high, especially among patients with significant comorbidities or those who develop complications such as multiple organ failure. This elevated mortality is frequently attributed to delayed or inaccurate diagnosis, often resulting from misidentification of the pathogen as other fungi. Furthermore, the organism’s capacity to cause severe infections, combined with challenges in diagnostic accessibility and the current lack of understanding regarding its resistance mechanisms, likely contributes to the observed high fatality rates.

This study is subject to several limitations. The literature search may not have captured all relevant data on epidemiology and mortality, as some studies could have been overlooked due to the search strategy used. Our analysis relied solely on case reports and case series, which are dependent on accurate documentation for reliability. Moreover, many studies did not utilize molecular identification methods such as genetic sequencing, increasing the potential for misidentification. Incomplete reporting in some studies also limited the depth of our analysis, restricting it to data that were fully available. Lastly, excluding non-English language studies may have introduced a degree of selection bias, although the number of such articles was small. Additionally, this review is subject to publication bias, as rare or severe cases are more likely to be reported in the literature, potentially overestimating severity or mortality. The exclusion of non-English studies may have also contributed to language bias, possibly limiting the geographic and clinical diversity of included cases.

## 5. Conclusions

This review offers a comprehensive examination of the epidemiology, clinical manifestations, microbiological characteristics, antifungal susceptibility profiles, therapeutic approaches and clinical outcomes associated with fungemia caused by *Exophiala* species. Particular emphasis is placed on elucidating the pathogenic potential of this understudied genus. Among the identified species, *E. dermatitidis* emerged as the most frequently isolated, with fever being the predominant clinical presentation. The pathogen generally demonstrated resistance to several antifungal agents. Although standardized treatment guidelines are currently unavailable, voriconazole was the most commonly used antifungal across the reported cases, but the limited number of patients and heterogeneity in clinical management preclude any conclusions regarding its efficacy. Timely initiation of antifungal therapy, ideally guided by in vitro susceptibility testing, is imperative. Given the opportunistic nature of *Exophiala* spp. and the diagnostic difficulties arising from limitations in commercial identification systems, heightened awareness among clinicians and microbiologists is essential for accurate recognition and management. While this review is subjected to certain limitations, it underscores the need for future longitudinal and controlled studies to advance the understanding of *Exophiala* infections and to form evidence-based therapeutic strategies.

## Figures and Tables

**Table 1:** Characteristics of patients with *Exophiala* species fungemia.

Characteristic	All Patients (*n* = 32)	Survived (*n* = 21)	Died (*n* =11)
Age, years, median	58	59.5	57
Male gender, *n* (%)	21 (65.6)	12 (57.1)	9 (81.8)
Predisposing factors			
Central venous catheter, *n* (%)	31 (96.9)	21 (100)	10 (90.9)
Malignancy, *n* (%)	25 (78.1)	17 (81)	8 (72.7)
Immunosuppression, *n* (%)	10 (31.3)	6 (28.6)	4 (36.4)
Neutropenia, *n* (%)	8 (25)	5 (23.8)	3 (27.3)
HCT, *n* (%)	4 (12.5)	1 (4.8)	3 (27.3)
TPN, *n* (%)	4 (12.5)	2 (9.5)	2 (18.2)
Polymicrobial infection, *n* (%)	5 (15.6)	4 (19)	1 (9.1)
Clinical characteristics			
Fever, *n* (%)	16 (50)	9 (42.9)	7 (63.6)
Organ dysfunction, *n* (%)	7 (21.9)	1 (4.8)	6 (54.5)
Treatment			
Voriconazole, *n* (%)	20 (62.5)	15 (71.4)	5 (45.5)
Amphotericin B, *n* (%)	10 (31.3)	4 (19)	6 (54.5)
Micafungin, *n* (%)	9 (28.1)	3 (14.3)	6 (54.5)
Itraconazole, *n* (%)	6 (18.8)	4 (19)	2 (18.2)
Fluconazole, *n* (%)	4 (12.5)	3 (14.3)	1 (9.1)
Outcomes			
Deaths due to infection, *n* (%)	8 (25)	NA	NA
Deaths overall, *n* (%)	11 (34.4)	NA	NA

HCT: hematopoietic cell transplantation; TPN: total parenteral nutrition; NA: not applicable.

**Table 2:** Antifungal resistance rates excluding mold-active azoles.

Antifungal Agent	Number of Patients	Resistance (%)
Micafungin	9/9	100
Anidulafungin	4/5	80
Caspofungin	7/9	77.8
Fluconazole	5/10	50
Flucytosine	3/7	42.9
Amphotericin B	1/11	9.1

## Data Availability

Not applicable.

## Supplementary Materials

The following supporting information can be downloaded at https://www.mdpi.com/article/10.3390/pathogens14070706/s1, Table S1: Characteristics of included studies.
