# Supplementary material for: Exophiala Bloodstream Infections in Humans—A Narrative Review
Source: Pathogens. 2025 Jul 17;14(7):706. doi: 10.3390/pathogens14070706 (PMC12300604; doi:10.3390/pathogens14070706)
Supplement: Supplementary file 1 [file pathogens-14-00706-s001.zip › pathogens-3753774-supplementary.pdf]

Table S1. Characteristics of included studies.

Author name/ Reference number	Number of patients	Age (years)	Gender	Blood Culture	CVC culture	MALDI-TOF	DNA sequencing	Other Diagnostic Method
Mpakosi et al. [4]	1	0*	M	+	-	-	+	-
Kumar et al. [14]	1	0.16	F	+	+	-	+	-
Nakatani et al. [21]	1	0.16	F	+	-	-	+	-
Al-Obaid et al. [18]	1	3	M	+	+	-	+	-
Nachman et al. [20]	1	3	M	+	+	NR	NR	-
Maraki et al. [11]	1	4.5	M	+	+	+	-	-
Kabel et al. [22]	1	5	M	+	+	NR	NR	-
Hagiya et al. [17]	1	29	M	+	-	+	-	Pleural Fluid Culture
Guarro et al. [19]	1	37	M	+	+	NR	NR	-
Watanabe et al. [7]	1	45	M	+	-	+	-	-
Tzar et al. [12]	1	50	M	+	-	NR	NR	-
Simpson et al. [8]	1	53	F	-	+	NR	NR	-
Vasquez et al. [23]	14	56	F	+	+	NR	NR	-
Chalkias et al. [6]	1	57	M	+	-	NR	NR	-
Vasquez et al. [23]	14	57	M	+	+	NR	NR	-
Vasquez et al. [23]	14	58	F	-	+	NR	NR	-
Vasquez et al. [23]	14	61	F	+	+	NR	NR	-
LaRocco et al. [9]	1	61	F	+	-	NR	NR	-
Vasquez et al. [23]	14	62	M	+	+	NR	NR	-
Vasquez et al. [23]	14	63	M	+	+	NR	NR	-
Yoshida et al. [15]	1	69	F	+	-	+	-	-
Vasquez et al. [23]	14	69	M	+	+	NR	NR	-
Vasquez et al. [23]	14	69	F	+	+	NR	NR	-

Vasquez et al. [23]	14	73	M	+	+	NR	NR	-
Vasquez et al. [23]	14	74	F	+	+	NR	NR	-
Itoh et al. [13]	1	75	M	+	+	+	-	-
Vila et al. [16]	1	75	M	+	+	+	-	-
Vasquez et al. [23]	14	77	M	+	+	NR	NR	-
Vasquez et al. [23]	14	78	M	+	+	NR	NR	-
Vasquez et al. [23]	14	79	M	-	+	NR	NR	-
Vasquez et al. [23]	14	81	F	+	+	NR	NR	-
Ahamad et al. [10]	1	NR	F	+	+	+	-	-

Supplementary Table S1. Diagnostic approaches in increasing patients' age order.

**: neonate, CVC: Central Venous Catheter, M: Male, F: Female, NR: Not Reported*
